# Seroprevalence of community-acquired atypical bacterial pneumonia among adult COVID-19 patients from a single center in Al Madinah Al Munawarah, Saudi Arabia: A retrospective cohort study

**DOI:** 10.15537/smj.2022.43.9.20220379

**Published:** 2022-09

**Authors:** Sari T. Alhoufie, Nadir A. Ibrahim, Naif H. Alsharif, Khalid O. Alfarouk, Hatim M. Makhdoom, Khaled R. Aljabri, Sayed H. Saeed, Adnan A. Khoumaeys, Yahya A. Almutawif, Mustafa A. Najim, Hamza M. Ali, Alanoud A. Aljifri, Ali M. Kheyami, Areej A. Alhazmi

**Affiliations:** *From the Medical Laboratories Technology Department (Alhoufie, Ibrahim, Makhdoom, Almutawif, Najim, Ali, Alhazmi), College of Applied Medical Sciences, Taibah University; from the Department of Medical laboratory,(Alsharif, Saeed, Aljabri, khoumaeys) king Salman Medical City, Al Madinah General hospital, from Al-Madinah Health Cluster(Aljifri and Kheyami, Ministry of Health Madinah Al Munwarah, Kingdom of Saudi Arabia; and from the Institute of Endemic Diseases (Alfarouk), University of Khartoum, Khartoum, Sudan.*

**Keywords:** SARS-COV-2, community-acquired bacterial pneumonia, *Chlamydia pneumonia*, *Mycoplasma pneumoniae*, *Legionella pneumophila*

## Abstract

**Objectives:**

To investigate the seroprevalence of the community-acquired bacterial that causes atypical pneumonia among confirmed severe acute respiratory syndrome coronavirus 2 (SARS-COV-2) patients.

**Methods:**

In this cohort study, we retrospectively investigated the seroprevalence of *Chlamydia pneumoniae*, *Mycoplasma pneumoniae*, and *Legionella pneumophila* among randomly selected 189 confirmed COVID-19 patients at their time of hospital presentation via commercial immunoglobulin M (IgM) antibodies against these bacteria. We also carried out quantitative measurements of procalcitonin in patients’ serum.

**Results:**

The seropositivity for *L. pneumophila* was 12.6%, with significant distribution among patientsolder than 50 years (χ2 test, *p*=0.009), while those of *M. pneumoniae* was 6.3% and *C. pneumoniae* was 2.1%, indicating an overall co-infection rate of 21% among COVID-19 patients. No significant difference (χ2 test, *p*=0.628) in the distribution of bacterial co-infections existed between male and female patients. Procalcitonin positivity was confirmed amongst 5% of co-infected patients.

**Conclusion:**

Our study documented the seroprevalence of community-acquired bacteria co-infection among COVID-19 patients. In this study, procalcitonin was an inconclusive biomarker for non-severe bacterial co-infections among COVID-19 patients. Consideration and proper detection of community-acquired bacterial co-infection may minimize misdiagnosis during the current pandemic and positively reflect disease management and prognosis.

Since the emergence of the SARS-CoV-2 in Wuhan, China, in December 2019, rising incidence rates have been reported globally. Most cases result in only mild infections, while other patients with moderate infections might experience symptoms such as fever, shortness of breath, and fatigue. Severe infection can lead to pneumonia, respiratory failure, and acute respiratory distress syndrome.^1-3^

Incidence rates of co-infecting bacterial pathogens during previous emerging respiratory viral infection pandemics and endemics have been reported in the last few decades. For example, during the SARS-CoV-1 endemic in China (2003), a cohort study confirmed that more than 20% of SARS-CoV-1 patients were co-infected with bacterial pathogens.^4^ Additionally, bacterial pneumonia caused 29-55% of deaths during the N1H1 influenza pandemic in 2009.^5^ A retrospective cohort study in Saudi Arabia in 2017 also documented bacterial co-infections among 18% of critically ill patients infected with the Middle East respiratory syndrome coronavirus.^6^

As the COVID-19 pandemic continues, a similar phenomenon of bacterial co-infection has also been observed. In China, an early study revealed bacterial co-infection among severely ill COVID-19 patients.^7^ Another observational study in Italy of 16,654 COVID-19 critically ill patients reported bacterial and fungal co-infections among 11% of the study population.^8^

Managing respiratory bacterial co-infection among COVID-19 patients is challenging in different aspects. For instance, more complex treatment precautions must be adopted to avoid administering medications that negatively interact with COVID-19 therapies. Moreover, bacterial co-infection may increase the severity of the patient’s illness and the mortality rate.^9,10^ Most bacterial co-pathogens found among COVID-19 patients are hospital-acquired, multidrug-resistant (MDR) bacteria such as *Enterococcus spp.*, *Acinetobacter baumannii*, *Pseudomonas aeruginosa*, methicillin-resistant *Staphylococcus aurfeus*, *Klebsiella spp.*, and infection often occurs after just a few (5-8) days of hospital admission.^11^ Most of those patients are critically ill COVID-19 patients. They are more likely to receive treatments involving invasive catheters, central lines, and auto-ventilation, which might increase the possibility of MDR bacteria co-infection.^11,12^

Meanwhile, cases of SARS-CoV-2 infection with bacteria capable of causing community-acquired pneumonia, such as *Chlamydia pneumoniae* (*C. pneumoniae*), *Mycoplasma pneumoniae* (*M. pneumoniae*), and *Legionella pneumophila* (*L. pneumophila*), have also been observed, albeit to a lesser degree than hospital-acquired bacterial pathogens.^12^ An early observational study of these atypical pneumonia-causing bacterial agents was carried out among critically ill COVID-19 patients in China, pointing out that *M. pneumoniae* and *L. pneumophila* are the most frequently identified pathogens.^13^

Procalcitonin (PCT) is a precursor of the hormone calcitonin containing 116 amino acids released and synthesized by thyroid parafollicular C cells in normal conditions. It can also be produced in many extra thyroid tissues and released into the bloodstream during severe bacterial infections, as the lipopolysaccharides in the cell walls of Gram-negative bacteria play a role in PCT triggering; therefore, it is one of the biomarkers used to investigate the existence of severe bacterial infections.^14^

Despite the ongoing research on bacterial co-infections among COVID-19 patients, little is known regarding the incidence rates of community-acquired pneumonia bacterial co-infections among COVID-19 patients in Saudi Arabia. Therefore, we carried out this retrospective cohort study to investigate their seroprevalence amongst these patients. We used enzyme-linked immunosorbent assay (ELISA) to detect the presence of immunoglobulin M (IgM) antibodies in the tested samples for *C. pneumoniae*, *M. pneumoniae*, and *L. pneumophila*, IgM antibody commonly used for the existence of acute infection. Therefore, seropositive samples would be an indicator of recent infection.

## Methods

In this retrospective study, a total of 189 serum samples were obtained from patients with confirmed SARS-CoV-2 infection (Rt-PCR confirmed) at the time of their presentation to the Emergency Department at Al-Madinah General Hospital, King Salman Medical City, Al-Madinah Al-Munawarah, before their hospitalization. Serum samples were collected from the serology section of the hospital clinical laboratory and aliquoted, then immediately stored at -20°C for further investigation. Samples were collected during the pandemic from May to December 2020. Al-Madinah General Hospital, Al-Madinah Al-Munawarah, is a tertiary care center that has been designated by the health authority of the Al-Madinah Al-Munawarah region to provide medical care for COVID-19 patients during the pandemic. The inclusion criteria were any confirmed SARS-CoV-2 patients with a hospital admission date of no more than 6 days. The exclusion criteria were: I) non confirmed COVID-19 patients; and II) confirmed COVID-19 patients with more than 6 days of hospital admission.

The Institutional Review Board of the General Directorate of Health Affairs in Al-Madinah Al-Munawarah, approved this study (approval no.: 9-2021). Patients’ data were extracted from their medical records.

### Serodetection of M. pneumoniae, Chlamydophila pneumoniae, and L. pneumophila

To assess the existence of atypical pneumonia-causing bacteria in the serum of COVID-19 patients, IgM antibodies against *M. pneumoniae* and *C. pneumoniae* were screened by an indirect ELISA method using Vircell IgM ELISA kits (Vircell, Granada, Spain), as instructed. Separately, the serum samples were screened for IgM antibodies against *L. pneumophila* serogroups 1-7 using Serion ELISA classic kits (Institut Virion\Serion GmbH, Würzburg, Germany) according to the manufacturer’s instructions. All steps were carried out at 37°C in a moist chamber. Before running the test, rheumatoid factor-absorbent was diluted in dilution buffer; then, samples were diluted 1:100 with rheumatoid factor-absorbent dilution buffer. One well was used for substrate blanking, and patient and control sera were incubated in microplates for 60 minutes. Next, the wells were washed and incubated with the IgM conjugate for 30 minutes. After a second round of washing, para-nitrophenyl phosphate substrate was added and incubated for 30 minutes. Then, stopping solution was added immediately into all wells, and the absorbance was measured at 405 nm. All washing steps were run using a semi-automated ELISA washer and reader (Biotek, Winooski, VT, USA) according to the manufacturer’s user manual. Assay tests were carried out in duplicate, and results were interpreted according to the instructions provided with the assay (lot no. SKH.BT).

### Quantitative determination of procalcitonin (PCT) concentrations

Quantitative measurements of PCT concentration in COVID-19 patients’ serums were applied to estimate the severity of bacterial co-infection. Procalcitonin concentration levels were measured using the QuicKey Human PCT Sandwich-ELISA Kit (Elabscience, Houston, TX, USA) per the manufacturer’s instructions. In brief, a standard working solution of 20 ng/mL was prepared; then, serial dilutions (20, 10, 5, 2.5, 1.25, 0.63, 0.31, and 0 ng/mL) were established. Next, 50 µL of each diluted standard and samples were added to the appropriate wells coated with PCT capture antibody, and all samples and standards were assayed in duplicate. A 50 µL of detection antibody solution was added to each well immediately, and the plate was covered and incubated for 90 minutes at 37°C. Wells were washed with 350 µL of washing buffer for 3 cycles, and 100 µL of horseradish peroxidase conjugate solution was subsequently added to each well and incubated for 30 minutes at 37°C. The washing process was then repeated 5 times. Next, the substrate was added, and the plate was incubated for 15 minutes at 37°C while being protected from light. Stop solution was added in the same order as the substrate solution. The optical density for each well was determined using an ELISA reader (Biotek) according to the manufacturer’s user manual. The duplicate readings for each standard and sample were averaged, and the average zero standard optical density was subtracted. Finally, a 4-parameter logistic standard curve was plotted to determine the concentrations of unknown samples with a cutoff value of 0.5 ng/mL or greater.

### Statistical analysis

Bacterial infection distributions concerning gender and age were compared using the Chi-squared (χ^2^) test. *P*-values of 0.05 were considered statistically significant. Data were analyzed using the Statistical Package for the Social Sciences, version 20 (IBM Corp., Armonk, NY, USA).

## Results

A total of 189 serum samples from patients with confirmed SARS-CoV-2 infection cases nasopharyngeal swabs RT-PCR results were assessed for IgM antibodies against atypical pneumonia-causing bacteria *M. pneumoniae*, *C. pneumoniae*, and *L. pneumophila*.

**Figure 1:**
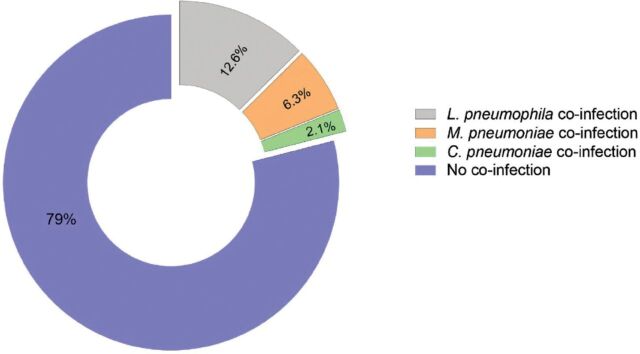
- Prevalence of sero-positivity for community acquired atypical pneumonia bacteria co-infections amongst 189 SARS-CoV-2 patients.

Seropositivity was confirmed in 40 (21%) patients, with *L. pneumophila* in 12.6%, *M. pneumoniae* in 6.3%, and *C. pneumoniae* in 2.1% infections confirmed of this group (Figure 1). A total of 125 (66.1%) patients were male, and 64 (33.9%) patients were female, and the distribution of bacterial co-infection cases according to the causative organism among males (101/152; 66.4%) and females (51/152; 33.6%) patients showed no significant difference (*p*=0.628).

**Figure 2:**
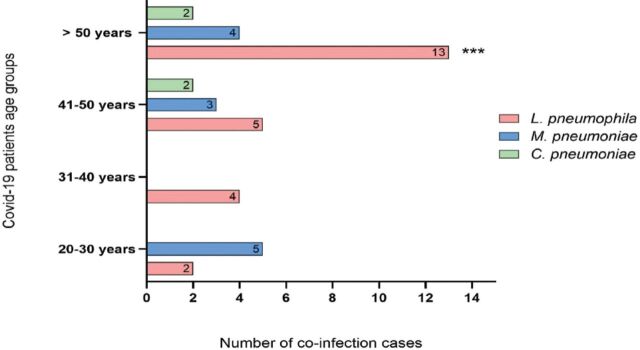
- Distribution of community acquired atypical pneumonia bacterial agents co-infections between different age groups of 189 SARS-CoV-2 patients. ***L. pneumophila co-infections cases were significant (χ2 test, *p*=0.009) among >50 years patients in comparison with other age groups.

The mean age of study participants was 51 years, and they were divided into 4 groups according to age: 20-30 years, 31-40 years, 41-50 years, and older than 50 years. Co-infection with *Legionella* was confirmed in all age groups and was the most common co-infection (13/24; 54.2%) among those older than 50 years (*p*=0.009). Considering *Mycoplasma*, the data revealed cases of co-infection existed in all age groups except those aged 20-30 years, with no significant difference in distribution (*p*=0.779). *Chlamydophila* cases were underrepresented in our data and were detected only among those older than 41 years, with no significant difference (*p*=1; Figure 2).

Despite the seropositivity findings in our 40 patients’ serum for the 3 bacterial agents of interest, negative PCT detection results were acquired for most co-infected patients (38/40) as their PCT level was less than 0.5 ng/mL. Only 2 patients infected with *M. pneumoniae* and *L. pneumophila* had positive results, as their PCT levels were 0.7 and 3.8 ng/mL. However, among the total 189 COVID-19 cases in this study, 2 patients did not survive, and their serum was positive for both *M. pneumoniae* and *L. pneumophila* with negative PCT results.

## Discussion

In this study, we carried out a retrospective cohort study to investigate IgM seropositivity for *M. pneumoniae*, *C. pneumoniae*, and *L. pneumophila*, which are community-acquired bacterial pathogens capable of causing atypical pneumonia, among randomly selected COVID-19 patients seen at a single tertiary care center. This study was carried out between May and December 2020, where more than 362,741 cases and at least 6,223 deaths had occurred in Saudi Arabia.^15^

We showed a seropositivity (IgM) rate for these atypical bacteria, which indicated current or acute co-infection in 40 (21%) of the 189 COVID-19 patients observed in this study (Figure 1). *Legionella pneumophila* was the most common bacterial co-pathogen, detected in 60% of co-infected COVID-19 patients, and with an incidence that was increased significantly among COVID-19 patients older than 50 years (Figure 2). *Mycoplasma pneumoniae* occurred in 30% and *C. pneumoniae* occurred in 10% of the cases, and no significant difference in co-infection incidence rates was determined according to their gender.

However, before the current pandemic, an early etiological diagnosis (1992) on community-acquired pneumonia in the central region of Saudi Arabia found that 62% of pneumonia patients infected with community-acquired bacterial pathogens, *Streptococcus pneumoniae* (26%) and *M. pneumoniae* (23%) were the most frequent agents.^16^ Further observation in 2011 by Asghar and his research group reported community-acquired atypical pneumonia during the Hajj season in Makkah, Saudi Arabia; they found the seroprevalence of *L. pneumophila* was 14.9% among pneumonia patients from 3 leading tertiary care hospitals.^17^ Balkhy et al^18^ claimed that community-acquired pneumonia is the most frequent community-acquired infection, and it is highly prevalent among those aged 50 years and older, which is in concur with our finding here.

Few studies to date have reported on co-infections with community-acquired bacteria capable of causing atypical pneumonia among COVID-19 patients, a trend which may be due to undervaluing the assessment of respiratory co-infections during the emergence of a novel viral pathogen.^19^ Furthermore, similar clinical manifestations, such as fever, chest pain, and dyspnea, may appear with atypical pneumonia caused by bacterial infection and SARS-CoV-2 infection, which could play a role in misdiagnosing the former.^20,21^ In addition, the adoption of routine techniques for culturing blood and respiratory tract samples may only detect 5-10% of causative pathogens in pneumonia patients.^22^

Despite the underdiagnosed bacteria co-infections present among COVID-19 cases in this study, most of our patients (n=187) survived. It is worth noting that all patients received empirical treatment, including azithromycin, under the current treatment guidelines for COVID-19 patients. Empirical treatment coverage, including macrolide for atypical bacteria, is efficient for hospitalized adult patients with atypical pneumonia caused by community-acquired bacteria.^23,24^ The administration of azithromycin might have reduced the elevation of COVID-19 severity caused by bacterial co-infection.

Procalcitonin detection was carried out to investigate the existence of severe bacterial infections in all patients yet confirmed *M. pneumoniae* and *L. pneumophila* co-infections in only 2 COVID-19 patients. Negative PCT results might be obtained as confirmation of no severe bacterial infection. Still, it is essential to consider that interferon-gamma is released during viral infections, including SARS-CoV-2 infection. Studies suggest this cytokine shows significant obstructive activity in the PCT synthesis pathway that might cause a low PCT level in patients despite a bacterial co-infection.^25^ This finding agrees with the recent observational study by May et al^26^ who found low levels of PCT among COVID-19 patients with community-associated bacterial co-infection. In contrast, the PCT level was increased significantly among non-COVID-19 patients infected with community-associated bacteria.^26^ Therefore, PCT appears to be an unreliable biomarker for ruling in/out bacterial co-infection and determining when to initiate antibiotics treatments for non-severely ill COVID-19 patients with bacterial co-infection.

Of interest, a recent study of 48 COVID-19 patients in Saudi Arabia reported 27 cases of viral con-infection co-infection including influenza and human adenovirus, and 13 cases of *C. pneumoniae* co-infection among intensive care unit (ICU) and non-ICU patients.^27^ Still, it was not clear whether these cases were community-acquired or hospital-acquired co-infections. Early detection of bacteria capable of causing atypical pneumonia via serological and molecular approaches can determine the source of infection, which would help with the early management of an infected patient and hospital infection-control measurements in general.

### Study limitations

Besides the smaller sample size, this study was a single-center investigation. As a retrospective observational study, there are limitations in the diagnostic tests obtained and procedures carried out at the time of clinical care. A lack of details in some patients’ clinical records, such as comorbidities, radiography and laboratory results, and the full panel of treatments, is another limitation that should be avoided in prospective studies in this field.

In conclusion, our study highlights the seroprevalence of co-infection with community-acquired bacteria capable of causing atypical pneumonia among COVID-19 patients at the time of hospital admission in Saudi Arabia. The overlap in clinical manifestations between bacterial and SARS-CoV-2 infections may increase the rate of misdiagnosis of the former, which might negatively influence the patient’s management and prognosis.
